# Effect of Apixaban Pretreatment on Alteplase-Induced Thrombolysis: An *In Vitro* Study

**DOI:** 10.3389/fphar.2021.740930

**Published:** 2021-09-15

**Authors:** Sandra Thalerová, Michaela Pešková, Patrícia Kittová, Sumeet Gulati, Jan Víteček, Lukáš Kubala, Robert Mikulík

**Affiliations:** ^1^ Neurology Department, International Clinical Research Center, St. Anne’s University Hospital Brno, Brno, Czechia; ^2^ Institute of Biophysics of the Czech Academy of Sciences, Brno, Czechia; ^3^ Department of Biochemistry, Faculty of Science, Masaryk University, Brno, Czechia; ^4^ Center of Biomolecular and Cell Engineering, International Clinical Research Center, St. Anne’s University Hospital Brno, Brno, Czechia

**Keywords:** alteplase, apixaban, clot, *in vitro*, thrombolysis, stroke

## Abstract

Benefit of thrombolytic therapy in patients with acute stroke, who are on anticoagulant treatment, is not well addressed. The aim of this study was to investigate whether apixaban can modify the thrombolytic efficacy of alteplase *in vitro*. Static and flow models and two variants of red blood cell (RBC) dominant clots, with and without apixaban, were used. Clots were prepared from the blood of healthy human donors and subsequently exposed to alteplase treatment. Apixaban and alteplase were used in clinically relevant concentrations. Clot lysis in the static model was determined both by clot weight and spectrophotometric determination of RBC release. Clot lysis in the flow model was determined by measuring recanalization time, clot length and spectrophotometric determination of RBC release. In the static model, clots without apixaban; compared to those with apixaban had alteplase-induced mass loss 54 ± 8% vs. 53 ± 8%, *p* = 1.00; RBC release 0.14 ± 0.04 vs. 0.12 ± 0.04, *p* = 0.14, respectively. Very similar results were obtained if plasma was used instead of physiological buffered saline as the incubation medium. In the flow model, clot lysis without apixaban; compared to those with apixaban was as follows: recanalization time 107 ± 46 min vs. 127 ± 31 min, *p* = 1.00; recanalization frequency 90 ± 22% vs. 90 ± 22%, *p* = 1.00; clot volume reduction 32 ± 15% vs. 34 ± 10%, *p* = 1.00; RBC release 0.029 ± 0.007 vs. 0.022 ± 0.007, *p* = 0.16, respectively. Apixaban had no positive effect on alteplase-induced thrombolysis in both the *in vitro* static and flow models. Our data support current clinical practice, such that thrombolysis is contraindicated in stroke treatment for patients who have been treated with anticoagulants.

## Introduction

Non-vitamin K antagonist oral anticoagulants [novel oral anticoagulants (NOACs)] are the preferable option over warfarin for secondary prevention of thrombotic events in patients with atrial fibrillation (Connolly et al., 2009; Granger et al., 2011; Patel et al., 2011). Their use in clinical practices is thus increasing (Jin et al., 2018; Chao et al., 2019). More frequent use, however, of NOACs itself poses new challenges in patients subsequently suffering from stroke. While on NOACs, thrombolytic treatment is usually contraindicated (Tsivgoulis and Safouris, 2017; Šaňák et al., 2018; Shahjouei et al., 2020; Tsivgoulis et al., 2021). The only alternative, mechanical thrombectomy, is however, suitable only for patients with large vessel occlusion and is not universally available (Šaňák et al., 2018; Shahjouei et al., 2020). Idarucizumab is an antidote that allows thrombolysis in patients on dabigatran (Šaňák et al., 2018). It is not, however, clear if the antidote for direct factor Xa (FXa) inhibitors, adexanate, will allow thrombolysis in patients on apixaban and/or if the cost of adexanate will prevent its use in many countries around the world. Therefore, it is important to study interaction between intravenous alteplase and NOACs, especially direct FXa inhibitors.

Apixaban is a NOAC, which directly inhibits both free and clot-bound coagulation factor FXa, preventing prothrombin cleavage to active thrombin (Jiang et al., 2009; Connolly et al., 2011; Byon et al., 2019). *In vitro* studies (Jiang et al., 2009; Escolar et al., 2013) have demonstrated that, by interfering with thrombin generation, apixaban prolongs clotting time and clot formation time, thereby altering viscoelastic parameters during clot formation. Animal models have also shown that apixaban reduces platelet and fibrin formations on damaged vessels (Escolar et al., 2013) and inhibits clot formation in a dose-dependent manner (Pinto et al., 2007). Recent studies (Farag et al., 2016; Carter et al., 2018; Spinthakis et al., 2019) have also indicated that apixaban shortens fibrinolysis times and enhances endogenous fibrinolysis in patients.

Despite the efficiency of apixaban pretreatment in high-risk stroke patients, its interaction with thrombolytics is not well known (Ishihara et al., 2014; Seiffge et al., 2015; Jin et al., 2018; Chao et al., 2019). In order to provide a better understanding, we established a combination of two *in vitro* thrombolytic models to investigate the interaction between apixaban pretreatment and alteplase-induced thrombolysis.

## Methodology

This is an *in vitro* study using both static and flow models to study the effect of apixaban pretreatment on alteplase-induced thrombolysis and recanalization using apixaban-pretreated human blood clots.

### Treatment Groups

Four experimental groups were established to test the hypothesis that apixaban-pretreatment with subsequent alteplase-treatment had different outcome, as compared to alteplase-treatment of clots. These four groups included: “untreated” (subject not on apixaban, without thrombolytic treatment), “alteplase-treated” (subject not on apixaban, with thrombolytic treatment), “apixaban-pretreated” (subject on apixaban, without thrombolytic treatment) and “apixaban-pretreated + alteplase-treated” (subject on apixaban, with thrombolytic treatment).

### *In Vitro* Model

Experiments were performed in static and flow *in vitro* models described in contemporary literature (Harpaz et al., 1993; Prasad et al., 2006), which were optimized to determine the suitably measurable effect of alteplase in highly repeatable manner. Briefly, the static model consisted of 1.5 ml plastic tubes (Eppendorf, Germany) filled with medium to a total volume of 500 μL, in which the clots were individually incubated. Tubes were placed into a dry-block incubator at 37°C and incubation lasted 60 min (the same amount of time indicated for alteplase treatment of stroke patients). The flow model comprised of silicone chips (Sylgard 184 Silicone Elastomer, Dow Corning, United States) prepared according to human middle cerebral artery anatomy, with narrowings dimensionally based on patient CT scans (*n* = 4). The bifurcation was included to enable permanent circulation in the system. Each silicone chip was connected by plastic pipes (internal diameter 3.1 mm) to an 8-channel pump head peristaltic pump (Gilson Minipuls 3, Gilson, Inc., United States) and the whole system was maintained at 37°C for incubation lasting 180 min. This arrangement maintained the hydrodynamic forces involved in clot removal (Diamond, 1999), whereby a pressure gradient of 10 mm Hg was generated across the occlusion*.*

### Clots and Media Preparation

RBC dominant clots were employed in both types of *in vitro* model. In the static model, clots were prepared from 200 μL whole human venous blood and clotted in borosilicate glass tubes (internal diameter 8 mm) for 5 h at room temperature to allow for proper retraction (Sutton et al., 2013). Clots were either incubated in physiological buffered saline (PBS, pH 7.4) or 5-fold diluted human plasma. Concerning the flow model, clots were prepared from 100 μL blood and clotted in borosilicate glass tubes (internal diameter 6 mm) for 4 h at room temperature. Clots in this instance were incubated in 5-fold diluted human plasma only.

Two clots variants were used–those without or; those supplemented with apixaban (250 ng ml^−1^), (a representative of the apixaban-pretreated group). Both variants were divided into two groups: untreated and alteplase-treated (1.3 mg L^−1^).

All blood donors had agreed to donate blood samples on the premise of signed informed consent for the collection of blood. Individuals who had received acetylsalicylic acid, non-steroidal anti-inflammatory or antiplatelet drugs within 7 days before blood collection were excluded.

All media were prepared using reagent grade chemicals. PBS (pH 7.4) contained 8 g NaCl, 2.3 g Na_2_HPO_4_.12H_2_O, 0.2 g KCl and 0.2 g KH_2_PO_4_ per liter. Plasma was freshly prepared for each experiment from donors’ citrated blood (in standard ratio 3.8% sodium citrate:blood, 1:9) by centrifugation (2000 g, 10 min, 4°C); diluted 5-fold with PBS and kept at 4°C prior to the experiment.

### Apixaban and Alteplase

Apixaban (provided by Pfizer Inc., United States; material no. 1151519, batch no. ABA8622) was initially dissolved in dimethyl sulfoxide, and subsequently diluted with PBS to a concentration of 2.5 mg ml^−1^. Diluted apixaban was stored aliquoted at −20°C and was not re-frozen once thawed. Concentration of apixaban was selected to be in line with clinically relevant dosing for the secondary prevention of thrombotic events in patients with atrial fibrillation, which is 250 ng ml^−1^ (Artang et al., 2017).

Alteplase (Actilyse, provided by Boehringer-Ingelheim International GmbH, Germany; Z. Nr. 1-24,717) was dissolved in distilled water to a concentration of 1 mg ml^−1^ and was stored aliquoted at -20°C (not re-frozen once thawed); after which it was further diluted with PBS. The final concentration of alteplase was also selected to be in line with clinically relevant dosing indicated for patients with ischemic stroke (1.3 mg L^−1^), according to the manufacturer’s instructions and supporting pharmacokinetic data (Acheampong and Ford, 2012).

### Measure of Lytic Efficacy

Clot lysis in the static model was determined by measuring clot weight (Prasad et al., 2006; Elnager et al., 2014) and by spectrophotometric determination of red blood cell (RBC) release into the incubation media at 575 nm. Clot lysis in the flow model was determined by measuring recanalization time in addition to clot length and spectrophotometric determination of RBC release. Recanalization frequency was determined as percentage ratio of complete recanalizations to the total number of samples in the given treatment group.

### Data Analysis and Statistics

We expected relative clot mass loss to be 30 ± 10% in the untreated group. In the static model, experiments in PBS were performed with two repetitions (with at least a 1-month interval) on samples from 10 healthy male blood donors (aged 31 ± 10 years, ranging from 22 to 53 years) for each variant of the clots (same donors for both clot variants), which would give us 80% power to detect relative clot mass loss of 43 ± 10% or more in the treated group. Experiments in 5-fold diluted plasma were performed on samples from 5 healthy male blood donors (mean age 32 ± 13 years, ranging from 23 to 53 years) for each variant of the clots (same donors for both clot variants). All samples were processed in triplicates.

We expected recanalization time to be 180 ± 25 min in the untreated group. Flow model experiments were performed on samples from 5 healthy male blood donors (mean age 32 ± 9 years, ranging from 23 to 53 years) for each variant of clots (different donors for clot variants), which would give us 80% power to detect recanalization time 135 ± 25 min or less in the treated group. All samples were processed in duplicates.

All analyses were performed with STATISTICA 12 (StatSoft) software. Data are expressed as mean ± SD and medians with minimum and maximum values. The forest plot shows mean values and confidence intervals (95%). Unpaired t-test was used to compare data. Bonferroni correction of *p*-value for multiple-comparisons was applied. The number of null hypotheses considered for static and flow model was 2 and 4, respectively. All *p*-values are reported after Bonferroni correction. *p*-values ≤ 0.05 were considered to be statistically significant.

## Results

### Thrombolysis

#### Static Model

**FIGURE 1:**
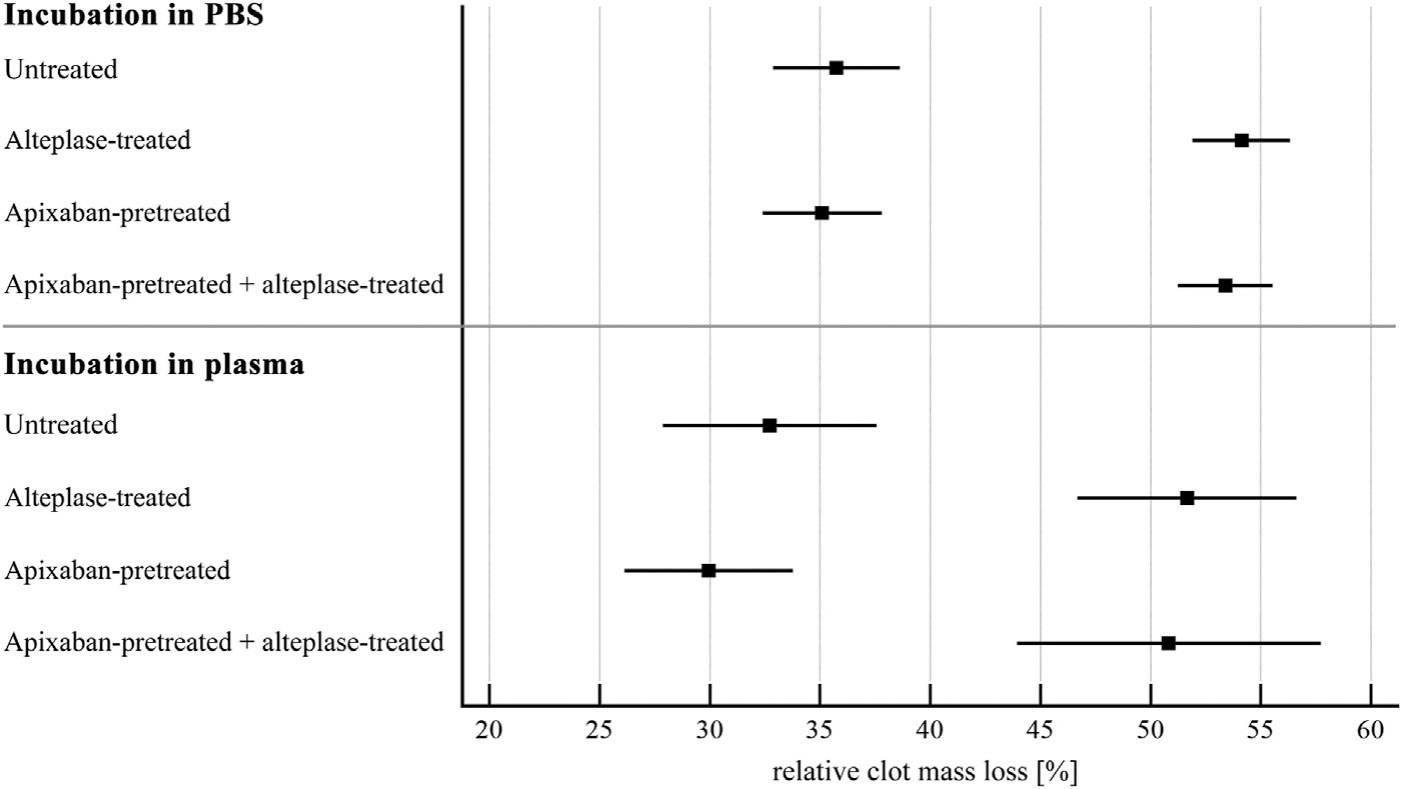
Static model: clot lysis expressed as relative clot mass loss. The forest plot shows mean values (square) and 95% confidence interval (whiskers). *n* = 54–57 for incubation in PBS and 12–15 for incubation in plasma. Results demonstrate that alteplase treatment provided efficient thrombolysis, e.g., documented as greater clot mass loss compared to untreated group in plasma (52 ± 9% vs. 33 ± 9%, *p* < 0.01). For clots with and without apixaban, alteplase-induced lysis did not differ, e.g., in plasma clot mass loss was 51 ± 11% vs. 52 ± 9%, *p* = 1.00. See Supplementary Table S1 for more details.

**FIGURE 2:**
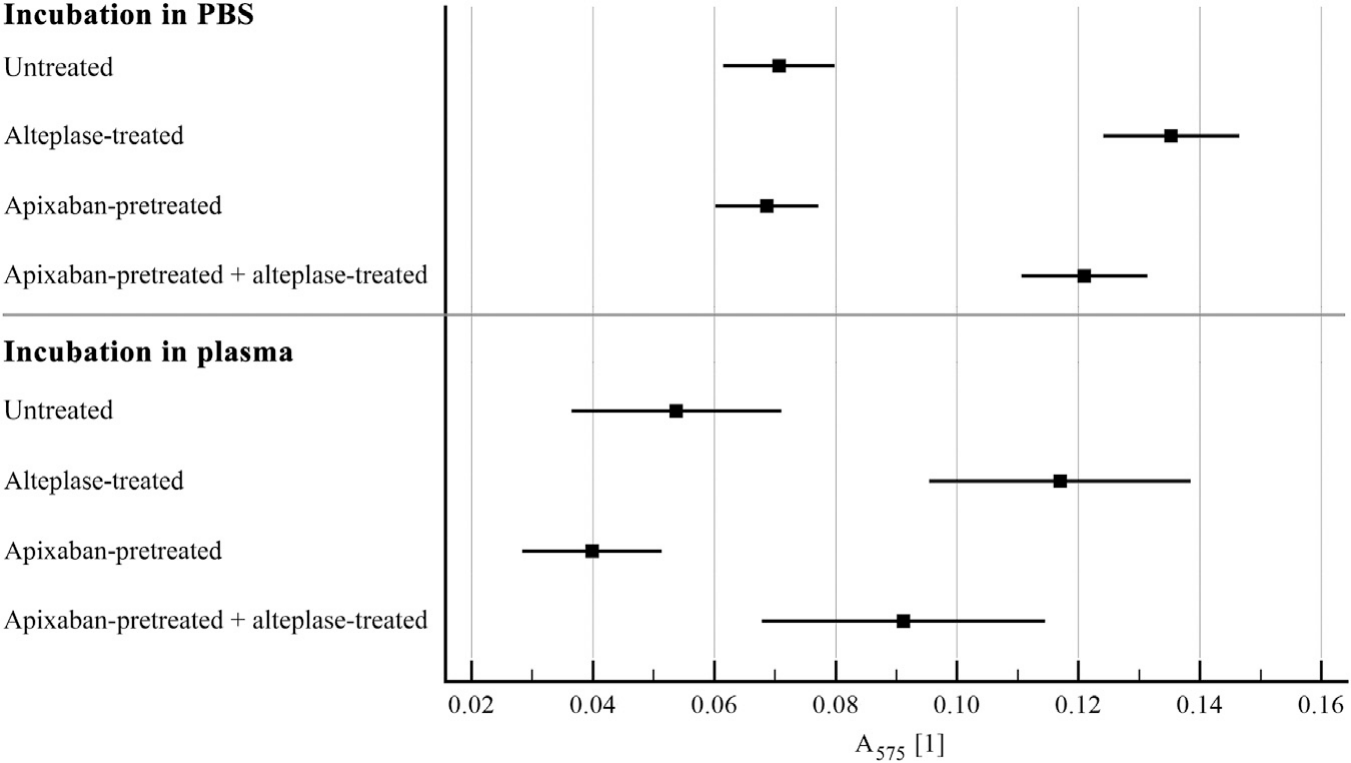
Static model: clot lysis expressed as red blood cell release into incubation media. The forest plot shows mean values (square) and 95% confidence interval (whiskers). *n* = 54–57 for incubation in PBS and 12–15 for incubation in plasma. Results demonstrate that alteplase treatment provided efficient thrombolysis, e.g., documented as greater red blood cell release compared to untreated group in plasma (0.12 ± 0.04 vs. 0.05 ± 0.03, *p* < 0.01). For clots with and without apixaban, alteplase-induced lysis did not differ, e.g., in plasma red blood cell release was 0.09 ± 0.04 vs. 0.12 ± 0.04, *p* = 0.18. See Supplementary Table S2 for more details.

Alteplase treatment provided greater clot mass loss compared to the untreated group (54 ± 8% vs. 36 ± 11%, *p* < 0.01) and greater RBC release compared to the untreated group (0.14 ± 0.04 vs. 0.07 ± 0.03, *p* < 0.01). For clots without apixaban and with apixaban, alteplase induced the same clot mass loss (54 ± 8% vs. 53 ± 8%, *p* = 1.00) and the same RBC release (0.14 ± 0.04 vs. 0.12 ± 0.04, *p* = 0.14). Additionally, clots without apixaban and with apixaban, both without alteplase treatment showed the same clot mass loss (36 ± 11% vs. 35 ± 10%, *p* = 1.00) and the same RBC release (0.07 ± 0.03 vs. 0.07 ± 0.03, *p* = 1.00). Results were nearly the same if plasma was used instead of PBS. Results are shown in Figures 1, 2 and in Supplementary Tables S1, S2.

#### Flow Model

**FIGURE 3:**
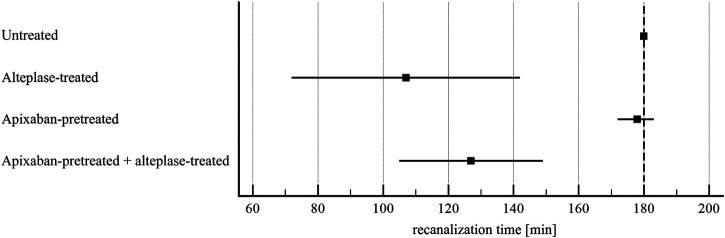
Flow model: time to recanalization of *in vitro* vessel. The forest plot shows mean values (square) and 95% confidence interval (whiskers). *n* = 9–10. Dashed line shows experiment time window. Results demonstrate that alteplase treatment provided efficient thrombolysis, e.g. documented as lower recanalization time compared to untreated group (107 ± 46 min vs. 180 ± 0 min, *p* < 0.01). For clots with and without apixaban, alteplase-induced lysis did not differ, e.g. recanalization time was 127 ± 31 min vs. 107 ± 46 min, *p* = 1.00. See Supplementary Table S3 for more details.

**FIGURE 4:**
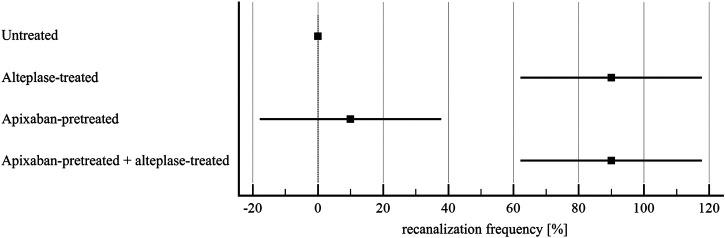
Flow model: recanalization frequency of *in vitro* vessel. The forest plot shows mean values (square) and 95% confidence interval (whiskers). *n* = 5. Results demonstrate that alteplase treatment provided efficient thrombolysis, e.g. documented as greater recanalization frequency compared to untreated group (90 ± 22% vs. 0 ± 0%, *p* < 0.01). For clots with and without apixaban, alteplase-induced lysis did not differ, e.g., recanalization frequency was 90 ± 22% vs. 90 ± 22%, *p* = 1.00. See Supplementary Table S3 for more details.

**FIGURE 5:**
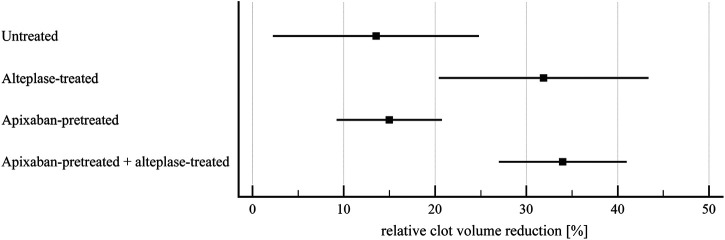
Flow model: clot lysis expressed as relative clot volume reduction. The forest plot shows mean values (square) and 95% confidence interval (whiskers). *n* = 9–10. Results demonstrate that alteplase treatment provided efficient thrombolysis, e.g., documented as greater clot volume reduction compared to untreated group (32 ± 15% vs. 14 ± 15%, *p* = 0.02). For clots with and without apixaban, alteplase-induced lysis did not differ, e.g., clot volume reduction was 34 ± 10% vs. 32 ± 15%, *p* = 1.00. See Supplementary Table S3 for more details.

**FIGURE 6:**
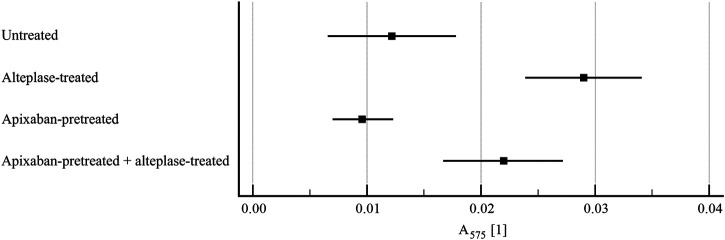
Flow model: clot lysis expressed as red blood cell release into incubation media. The forest plot shows mean values (square) and 95% confidence interval (whiskers). *n* = 9–10. Results demonstrate that alteplase treatment provided efficient thrombolysis, e.g. documented as greater red blood cell release compared to untreated group (0.029 ± 0.007 vs. 0.012 ± 0.007, *p* < 0.01). For clots with and without apixaban, alteplase-induced lysis marginally increased red blood cell release for clots without apixaban (0.022 ± 0.007 vs. 0.029 ± 0.007, *p* = 0.16). See Supplementary Table S3 for more details.

Alteplase treatment provided lower recanalization time compared to the untreated group (107 ± 46 min vs. 180 ± 0 min, *p* < 0.01) and greater recanalization frequency (90 ± 22% vs. 0 ± 0%, *p* < 0.01), RBC release (0.029 ± 0.007 vs. 0.012 ± 0.007, *p* < 0.01) and clot volume reduction (32 ± 15% vs. 14 ± 15%, *p* = 0.02). For clots without and with apixaban, alteplase induced the same recanalization time (107 ± 46 min vs. 127 ± 31 min, *p* = 1.00), recanalization frequency (90 ± 22% vs. 90 ± 22%, *p* = 1.00), clot volume reduction (32 ± 15% vs. 34 ± 10%, *p* = 1.00) and RBC release (0.029 ± 0.007 vs. 0.022 ± 0.007, *p* = 0.16). Results are shown in Figures 3–6 and in Supplementary Table S3.

### Clotting

**FIGURE 7:**
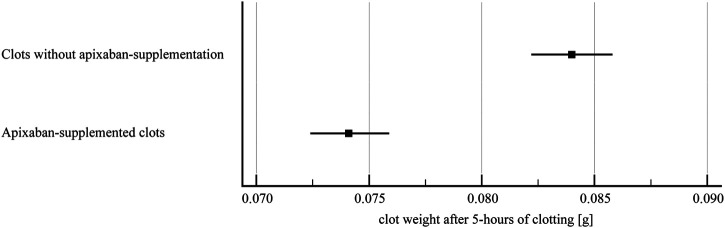
Clot weight after 5-h of clotting. The forest plot shows mean values (square) and 95% confidence interval (whiskers). *n* = 142 (clots without apixaban), *n* = 137 (apixaban-supplemented clots). Results demonstrate that apixaban supplementation provided lower clot weight after 5-h of clotting compared to clots without apixaban (0.07 ± 0.01 g vs. 0.08 ± 0.01 g, *p* < 0.01).

Clots prepared from blood without apixaban showed significantly higher weight after 5-h of clotting compared to clots supplemented with apixaban (0.08 ± 0.01 g vs. 0.07 ± 0.01 g, *p* < 0.01), (Figure 7). We have not observed any significant impact of such change of clot mass and respective volume in controls as well as alteplase-treated variants.

## Discussion

Our *in vitro* study aimed to determine whether apixaban pretreatment modifies efficacy of thrombolytic treatment with alteplase. Two *in vitro* models were used. Both were optimized to detect the lytic effect of alteplase in a highly repeatable manner. The effect of alteplase was observed even with PBS as the incubation medium and efficacy of alteplase was thus intermediated by plasminogen inside the clot. Concentrations of alteplase (and apixaban) were calculated to reflect therapeutic doses used in humans (Acheampong and Ford, 2012; Artang et al., 2017). The extent of the effect of alteplase was comparable to the rate of clot lysis in humans. Thus clot volume reduction median was 33% at 180 min with alteplase in the flow model, which is similar to the median clot volume reduction of 32% (Kim et al., 2015) in humans. All these findings support that although our experiments were conducted *in vitro*, results may be relevant to clinical data.

The major finding is that apixaban at clinically relevant concentration did not change the level of efficiency of alteplase *in vitro*. In the static model no significant impact of apixaban on alteplase efficiency was observed. Alteplase lysed apixaban-pretreated clots behaved similarly to clots without apixaban pretreatment, both in terms of clot mass loss and RBC release. Spontaneous thrombolysis of these clots remained similar as well. Minor differences in the initial clot mass did not affect thrombolysis. We reproduced this experiment in the static model in the presence of diluted plasma, since apixaban is a highly hydrophobic compound and plasma proteins could interact with it (Carter et al., 2018). Results were unchanged as compared with PBS. This indicated that all factors important for the alteplase action were already present in the clot and the interaction of apixaban with alteplase was independent of plasma proteins in our experimental system. Compared to previous *in vitro* studies with NOACs (apixaban and rivaroxaban) (Varin et al., 2013; Carter et al., 2018), we have not observed enhancement of thrombolysis by apixaban. Such observation is largely attributable to the therapeutic level of alteplase (∼1 mg L^−1^), which disfavor the molecular basis of the positive effect of a NOAC on plasminogen activation by alteplase (see detailed discussion below).

More importantly, in the flow model there was no influence of apixaban on alteplase efficiency as documented by four previously mentioned metrics: recanalization time, recanalization frequency and clot volume reduction and RBC release. Apixaban alone did not affect these characteristics as well. The obtained data are in agreement with the more simplistic static model. Compared to the static model, the flow model reflected clinical scenario better, because it included hemodynamic factors. Expectation was that the hydromechanical forces and resulting interstitial flow present in this system should allow better penetration of alteplase into the clot as previously reported (Diamond, 1999). To our knowledge there was no previous study documenting how apixaban can influence alteplase efficiency in an *in vitro* flow model, though the anticoagulant properties of apixaban were assayed under flow conditions in recent studies (Hosokawa et al., 2014; Sugihara et al., 2016; Pujadas-Mestres et al., 2017).

Previous experimental data, both *in vitro* and clinically indicated a positive effect of NOACs on thrombolysis, which is contrary to our finding. These studies however used alteplase at the endogenous level (∼1 μg L^−1^) (Varin et al., 2013; Carter et al., 2018) or studied impact of NOACs on endogenous thrombolysis (Farag et al., 2016; Spinthakis et al., 2019). The therapeutic dosage, however, resulted in concentration which is about three orders of magnitude higher (1.3 mg L^−1^ in this study). Massive activation of plasminogen by such high concentration of alteplase can overwhelm NOAC-mediated enhancement of slow initial activation of plasminogen on intact fibrin fibers (Talbot et al., 2013; Carter et al., 2018). That is why NOACs can enhance thrombolysis at endogenous levels of alteplase, as observed in previous studies (Varin et al., 2013; Farag et al., 2016; Lau et al., 2016; Carter et al., 2018; Spinthakis et al., 2019); but is excluded at therapeutic level of alteplase as presented in this study. Accordingly, our finding that apixaban at clinically relevant concentration did not change the efficiency of alteplase *in vitro* corresponds with recent clinical meta-analyses. They indicated that apixaban pretreatment did not increase efficiency of alteplase in ischemic stroke patients. The combination of apixaban pretreatment with subsequent thrombolytic therapy with alteplase may be safe for selected patients; however, more data is needed to determine risk of bleeding (Tsivgoulis and Safouris, 2017; Shahjouei et al., 2020; Tsivgoulis et al., 2021).

A possible limitation of our study is that we used blood from healthy donors. In clinical practice patients suffer from comorbidities. With the exception of diabetes, these comorbidities do not directly affect function of alteplase (Zangerle et al., 2007). Hence, we do not expect major differences in results that would be obtained using blood from patients. Another limitation of the presented *in vitro* models is the inability to study intracranial bleeding, which would be a clinical concern if alteplase was administered to patients treated with apixaban.

In conclusion, our data from two different *in vitro* models, static and flow, consistently indicated no effect of apixaban on thrombolysis and recanalization induced by therapeutic levels of alteplase. We used different metrics and documented that alteplase had a suitably measurable effect, which supports the validity of our findings. Our data thus support current clinical practice that patients are not treated with alteplase if they were previously medicated with apixaban; due to no positive impact on thrombolysis and recanalization but uncertain risk of bleeding.

## Data Availability

The original data presented in the study are included in Supplementary Material, further inquiries can be directed to the corresponding author.

## Abbreviations

FXa, factor Xa; NOACs, novel oral anticoagulants; PBS, physiological buffered saline; RBC, red blood cell.
