# Supplementary material for: Effect of Apixaban Pretreatment on Alteplase-Induced Thrombolysis: An In Vitro Study
Source: Front Pharmacol. 2021 Sep 15;12:740930. doi: 10.3389/fphar.2021.740930 (PMC8479181; doi:10.3389/fphar.2021.740930)
Supplement: Supplementary file 1 [file DataSheet1.docx]

# Supplementary data: Tables

Table 1: Static model: descriptive statistics for clot lysis expressed as relative clot mass loss. Four experimental groups were established: “untreated” (subject not on apixaban, without thrombolytic treatment), “alteplase-treated” (subject not on apixaban, with thrombolytic treatment), “apixaban-pretreated” (subject on apixaban, without thrombolytic treatment) and “apixaban-pretreated + alteplase-treated” (subject on apixaban, with thrombolytic treatment).

|  | **Mean**  **[%]** | **Median**  **[%]** | **SD^[[1]](#footnote-1)^**  **[%]** | **CI^[[2]](#footnote-2)^ (95%)**  **[%]** | **Range**  **[%]** | **Minimum**  **[%]** | **Maximum**  **[%]** | **Count** |  |
| --- | --- | --- | --- | --- | --- | --- | --- | --- | --- |
| **Incubation in PBS** | | | | | | | | | |
| Untreated | 36 | 36 | 11 | 3 | 46 | 11 | 58 | 56 |  |
| Alteplase-treated | 54 | 54 | 8 | 2 | 34 | 33 | 68 | 57 |  |
| Apixaban-pretreated | 35 | 35 | 10 | 3 | 44 | 13 | 58 | 57 |  |
| Apixaban-pretreated + alteplase-treated | 53 | 53 | 8 | 2 | 38 | 32 | 70 | 54 |  |
| **Incubation in 5-fold diluted plasma** | | | | | | | | | |
| Untreated | 33 | 32 | 9 | 5 | 27 | 19 | 46 | 15 |  |
| Alteplase-treated | 52 | 49 | 9 | 5 | 29 | 40 | 69 | 14 |  |
| Apixaban-pretreated | 30 | 31 | 7 | 4 | 20 | 20 | 40 | 14 |  |
| Apixaban-pretreated + alteplase-treated | 51 | 52 | 11 | 7 | 35 | 30 | 65 | 12 |  |

Table 2: Static model: descriptive statistics for clot lysis expressed as red blood cells released into incubation media. Four experimental groups were established: “untreated” (subject not on apixaban, without thrombolytic treatment), “alteplase-treated” (subject not on apixaban, with thrombolytic treatment), “apixaban-pretreated” (subject on apixaban, without thrombolytic treatment) and “apixaban-pretreated + alteplase-treated” (subject on apixaban, with thrombolytic treatment).

|  | **Mean**  **[1]** | **Median**  **[1]** | **SD^[[3]](#footnote-3)^**  **[1]** | **CI^[[4]](#footnote-4)^ (95%)**  **[1]** | **Range**  **[1]** | **Minimum**  **[1]** | **Maximum**  **[1]** | **Count** |  |
| --- | --- | --- | --- | --- | --- | --- | --- | --- | --- |
| **Incubation in PBS** | | | | | | | | | |
| Untreated | 0.07 | 0.06 | 0.03 | 0.01 | 0.12 | 0.01 | 0.13 | 56 |  |
| Alteplase-treated | 0.14 | 0.14 | 0.04 | 0.01 | 0.19 | 0.06 | 0.24 | 57 |  |
| Apixaban-pretreated | 0.07 | 0.06 | 0.03 | 0.01 | 0.13 | 0.01 | 0.14 | 57 |  |
| Apixaban-pretreated + alteplase-treated | 0.12 | 0.12 | 0.04 | 0.01 | 0.19 | 0.04 | 0.23 | 54 |  |
| **Incubation in 5-fold diluted plasma** | | | | | | | | | |
| Untreated | 0.05 | 0.05 | 0.03 | 0.02 | 0.10 | 0.01 | 0.11 | 15 |  |
| Alteplase-treated | 0.12 | 0.10 | 0.04 | 0.02 | 0.14 | 0.07 | 0.20 | 14 |  |
| Apixaban-pretreated | 0.04 | 0.03 | 0.02 | 0.01 | 0.06 | 0.01 | 0.07 | 14 |  |
| Apixaban-pretreated + alteplase-treated | 0.09 | 0.08 | 0.04 | 0.02 | 0.09 | 0.05 | 0.14 | 12 |  |

Table 3: Flow model: descriptive statistics for recanalization time, recanalization frequency and clot lysis expressed as red blood cell release and relative clot volume reduction. Four experimental groups were established: “untreated” (subject not on apixaban, without thrombolytic treatment), “alteplase-treated” (subject not on apixaban, with thrombolytic treatment), “apixaban-pretreated” (subject on apixaban, without thrombolytic treatment) and “apixaban-pretreated + alteplase-treated” (subject on apixaban, with thrombolytic treatment).

|  | **Mean** | **Median** | **SD^[[5]](#footnote-5)^** | **CI^[[6]](#footnote-6)^ (95%)** | **Range** | **Minimum** | **Maximum** | **Count** |  |
| --- | --- | --- | --- | --- | --- | --- | --- | --- | --- |
| **Recanalization time [min]** | | | | | | | | | |
| Untreated | 180 | 180 | 0 | 0 | 0 | 180 | 180 | 9 |  |
| Alteplase-treated | 107 | 98 | 46 | 35 | 130 | 50 | 180 | 9 |  |
| Apixaban-pretreated | 178 | 180 | 8 | 6 | 25 | 155 | 180 | 10 |  |
| Apixaban-pretreated + alteplase-treated | 127 | 125 | 31 | 22 | 93 | 87 | 180 | 10 |  |
| **Recanalization frequency [%]** | | | | | | | | | |
| Untreated | 0 | 0 | 0 | 0 | 0 | 0 | 0 | 5 |  |
| Alteplase-treated | 90 | 100 | 22 | 28 | 50 | 50 | 100 | 5 |  |
| Apixaban-pretreated | 10 | 0 | 22 | 28 | 50 | 0 | 50 | 5 |  |
| Apixaban-pretreated + alteplase-treated | 90 | 100 | 22 | 28 | 50 | 50 | 100 | 5 |  |
| **Relative clot volume reduction [%]** | | | | | | | | | |
| Untreated | 14 | 17 | 15 | 11 | 40 | 0 | 40 | 9 |  |
| Alteplase-treated | 32 | 33 | 15 | 11 | 40 | 17 | 57 | 9 |  |
| Apixaban-pretreated | 15 | 18 | 8 | 6 | 20 | 0 | 20 | 10 |  |
| Apixaban-pretreated + alteplase-treated | 34 | 33 | 10 | 7 | 33 | 17 | 50 | 10 |  |
| **Red blood cell release [1]** | | | | | | | | | |
| Untreated | 0.012 | 0.011 | 0.007 | 0.006 | 0.026 | 0.001 | 0.027 | 9 |  |
| Alteplase-treated | 0.029 | 0.029 | 0.007 | 0.005 | 0.028 | 0.019 | 0.042 | 9 |  |
| Apixaban-pretreated | 0.010 | 0.011 | 0.004 | 0.003 | 0.010 | 0.004 | 0.014 | 10 |  |
| Apixaban-pretreated + alteplase-treated | 0.022 | 0.021 | 0.007 | 0.005 | 0.026 | 0.008 | 0.034 | 10 |  |

1. SD, standard deviation [↑](#footnote-ref-1)
2. CI, confidence interval [↑](#footnote-ref-2)
3. SD, standard deviation [↑](#footnote-ref-3)
4. CI, confidence interval [↑](#footnote-ref-4)
5. SD, standard deviation [↑](#footnote-ref-5)
6. CI, confidence interval [↑](#footnote-ref-6)
